# Using an evolutionary algorithm and parallel computing for haplotyping in a general complex pedigree with multiple marker loci

**DOI:** 10.1186/1471-2105-9-189

**Published:** 2008-04-11

**Authors:** Sang Hong Lee, Julius HJ Van der Werf, Brian P Kinghorn

**Affiliations:** 1 The Institute for Genetics and Bioinformatics, School of Environmental and Rural Science, University of New England, Armidale, New South Wales 2351, Australia; 2 National Institute of Animal Science, Rural Development Administration, Cheon An, 330-801, Korea

## Abstract

**Background:**

Haplotype reconstruction is important in linkage mapping and association mapping of quantitative trait loci (QTL). One widely used statistical approach for haplotype reconstruction is simulated annealing (SA), implemented in SimWalk2. However, the algorithm needs a very large number of sequential iterations, and it does not clearly show if convergence of the likelihood is obtained.

**Results:**

An evolutionary algorithm (EA) is a good alternative whose convergence can be easily assessed during the process. It is feasible to use a powerful parallel-computing strategy with the EA, increasing the computational efficiency. It is shown that the EA can be ~4 times faster and gives more reliable estimates than SimWalk2 when using 4 processors. In addition, jointly updating dependent variables can increase the computational efficiency up to ~2 times. Overall, the proposed method with 4 processors increases the computational efficiency up to ~8 times compared to SimWalk2. The efficiency will increase more with a larger number of processors.

**Conclusion:**

The use of the evolutionary algorithm and the joint updating method can be a promising tool for haplotype reconstruction in linkage and association mapping of QTL.

## Background

Haplotypes can give useful information about patterns of inheritance for genomic regions. For each region, the probability of sharing founder genes through segregation in a recorded pedigree can be estimated based on the haplotypes, i.e. identity by descent (IBD) probability based on linkage (e.g. [1-3]). The probability of sharing the genes from a common ancestor before the recorded pedigree can also be estimated based on the haplotypes, i.e. IBD probability based on linkage disequilibrium (LD) (e.g. [4-7]). Those probabilities derived from the haplotypes are essential information for linkage mapping and association mapping.

Since haplotypes would not be directly observed from genotypic data, they need to be inferred based on observed pedigree information and marker genotypes. This would often result in a large state space of possible haplotype configurations especially with general pedigrees and incomplete genotypic data for multiple markers. Exact likelihood methods using pedigree peeling [8], chromosome peeling [9] or a combination of both algorithms often have problems dealing with the large state space and therefore have difficulties in finding the optimal haplotypes.

Alternatively, combinatorial optimization algorithms can be used. These are able to deal with problems that are hard to solve in polynomial time (NP hard). One strategy used in such algorithms is to search for haplotype configurations that require a minimum number of recombination events [10-12] or no recombination events [13]. These approaches are rule-based and do not make any assumptions about genetic distances between markers. Another approach is statistically based and would search for haplotype configurations with the highest likelihood, given all observed variables and known marker distances [14-16].

A widely used statistical approach for haplotype reconstruction is simulated annealing (SA) [17] which has been implemented in the linkage software, SimWalk2 [15,18]. SimWalk2 uses a random walk approach [19] to find candidates and an annealing process to develop the consecutive solutions to reach the optimal haplotypes. SimWalk2 constitutes a flexible and efficient algorithm for haplotyping and probably the only one used for a general complex pedigree with incomplete genotypes. However, it needs a very large number of sequential evaluations and it is not always guaranteed that the most likely solutions are found within the arbitrarily determined number of evaluations.

Evolutionary algorithms (EA) [20] constitute an efficient tool for solving combinatorial optimization problems. A number of parallel solutions are respectively updated (evolved) by changing the variables within each solution (EA-mutation), or recombining them from different solutions (EA-recombination), and the most favorable solutions are selected (EA-selection). Compared to SA, EA may be competitive in efficiently finding an optimal solution. An important advantage of EA is its potential to parallellise computations because the algorithm can be divided across multiple CPUs. This would substantially reduce computing time. Moreover, the search mechanism in EA can make it easier to diagnose convergence compared to that in SimWalk2. It is well known that EA can be easily designed and parameterised for a specific problem, and standard values for EA-parameters usually give reasonably good results [21].

In addition, jointly updating the set of dependent variables can increase the computational efficiency although not all sets of dependent variables may be found. It is noted that SimWalk2 attempts to update multiple variables together, but the set that is updated is randomly chosen (random walk approach). Because dependent variables are not necessarily within the same set, the acceptance rates are generally low.

An evolutionary algorithm has not been implemented before in statistical approaches for haplotyping. The aim of this study is to investigate the use of an evolutionary algorithm and joint updating strategy for haplotyping, and compare its efficiency with SimWalk2.

## Results

### Likelihood pattern with simulated data

Table 1 compares the likelihood of the best haplotypes found by SimWalk2, EA1 and EA2 for consecutive numbers of likelihood evaluation when using simulated data with 10 multiallelic markers positioned at 10 cM intervals. The results are averaged over 10 replicates (each result for each replicate is shown in additional file 1). When using complete genotypic data, the values for SimWalk2, EA1 and EA2 are not yet converged after 100,000 evaluations. The likelihood values for SimWalk2, EA1 and EA2 start to converge after 500,000 evaluations, and the values for EA1 and EA2 are higher than the value for SimWalk2. After 16,000,000 evaluations (this value is from the default annealing schedule of SimWalk2), the likelihood values may reach the global or nearly global maximum where the values for EA1 and EA2 are slightly higher than the value for SimWalk2. When comparing EA1 and EA2, the values are very similar after apparent convergence.

**Table 1:** Log likelihood of the best haplotypes found by SimWalk2, EA1 and EA2 with simulated data

	Complete genotypic data			
	
no. evaluations^‡^:	20000	100000	500000	16000000*
SimWalk2	-1960.81	-1827.53	-1804.93	-1801.7 (32 minutes)^¶^
EA1	-2197.17	-1852.43	-1802.16	-1800.55 (9 minutes)
EA2	-2299.78	-1848.51	-1802.39	-1800.55 (10 minutes)

	Incomplete genotypic data			
	
no. evaluations^‡^:	40000	400000	4000000	16000000

SimWalk2	-943.83	-791.41	-769.97	-767.43 (12 minutes)
EA1	-1108.24	-791.18	-766.74	-764.66 (6 minutes)
EA2	-1112.86	-782.19	-765.59	-764.43 (7 minutes)

^‡^number of evaluations of the likelihood function, *16000000 is from the default annealing schedule of SimWalk2

^¶^Computing time for SimWalk2, EA1 and EA2 (EA-population size N = 10 with 4 CPUs)

When using incomplete genotypic data, the likelihood values are not yet converged after 40,000 evaluations. After 400,000 evaluations, the likelihood values appear to be fairly close to the global maximum where the value for EA2 is the highest among the three methods (the values for EA1 and SimWalk2 are similar). After 4,000,000 evaluations, the likelihood reaches stable values with apparent convergence where the value for EA2 is the highest and the value for SimWalk2 is the worst. It is noted that the convergence patterns and the evaluation numbers are different between complete and incomplete genotypes.

### Likelihood pattern with real data

Table 2 shows the likelihood of the best haplotypes found by SimWalk2, EA1 and EA2 for consecutive numbers of likelihood evaluation when using the real data. The likelihood pattern for three methods is similar to that with simulated data however it is more clearly shown that EA2 outperforms SimWalk2 and EA1. After 520,000 evaluations, the configurations found by SimWalk2, EA1 and EA2 are far from the global maximum. The likelihood starts to reach stable values after 1,050,000 evaluations where the likelihood value for EA2 is higher than the values for EA1 and SimWalk2. It is shown that SimWalk2 gets a similar value at the evaluation number of 2,100,000 (Table 2). After 2,100,000 evaluations, the likelihood values are converged for three methods, and EA2 gives the highest value.

**Table 2:** Log likelihood of the best haplotypes found by SimWalk2, EA1 and EA2 with real data

No. evaluations:	260000	520000	1050000	2100000	21000000
SimWalk2	-24439.02	-23551.84	-23312.14	-23193.18	-23159.19 (434 minutes)^¶^
EA1	-25992.16	-24258.61	-23368.69	-23164.58	-23158.60 (106 minutes)
EA2	-25900.78	-23871.49	-23194.87	-23158.39	-23158.39 (126 minutes)

^¶^Computing time for SimWalk2, EA1 and EA2 (EA-population size N = 10 with 4 CPUs)

### Computing time for a fixed number of evaluations with 4 processors

The relative computing time between SimWalk2, EA1 and EA2 with 4 processors varies depending on data structure. When using simulated data with complete genotypes, the computing time completing 16,000,000 evaluations for EA1 and EA2 was 3.5 times and 3.2 times faster than SimWalk2 (Table 1). However, when using simulated data with incomplete genotypes, the relative computing efficiency for EA was decreased; EA1 was 2 times and EA2 was 1.7 times faster than SimWalk2 (Table 1). When using the real data, the computing time completing 21,000,000 evaluations for EA 1 and EA2 was 4.1 times and 3.5 times faster than SimWalk2 (Table 2). The larger advantage for EA for a larger data set is probably due to the fact that the proportion of transferring time over whole computing time increases when using a data set of small size with a few genotypes, e.g. simulated data with incomplete genotypes.

### Convergence diagnosis and computational efficiency

**Figure 1:**
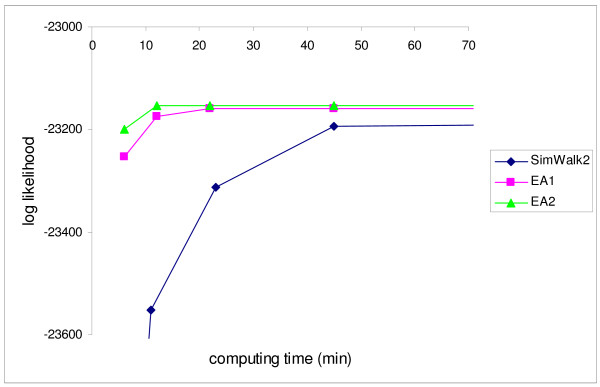
**Convergence and computational efficiency**. Log likelihood pattern of SimWalk2, EA1 and EA2 along with the actual computing time when using 4 processors simultaneously

Figure 1 shows the likelihood pattern of SimWalk2, EA1 and EA2 plotted against the actual computing time with the real data when using 4 processors simultaneously. EA2 reaches its maximum at ~10 minutes with an apparent convergence afterwards. EA1 reaches a similar likelihood value at ~20 minutes and shows a clear convergence. However, SimWalk2 is slower to reach convergence and still improving the likelihood. As shown in Figure 1, the computational efficiency for EA2 is up to ~2 times greater than EA1, and up to ~8 times greater than SimWalk2.

## Discussion

The EA1 and EA2 reach the global or nearly global maximum quicker than SimWalk2 both with simulated and real data. This was probably due to the fact that EA1 and EA2 used a number of parallel configurations which apparently is a more efficient searching mechanism, resulting in a wider range of variables updated during the cycles. Using an efficient joint updating strategy combined with the random walk, EA2 significantly outperformed SimWalk2 with a simultaneous use of multiple processors.

Convergence for EA1 (EA2) or SimWalk2 can be assessed by comparison of the likelihood values between different parts to check if the likelihood reaches a stable value (e.g. Figure 1). When the likelihood value is not converged, SimWalk2, as currently implemented, requires another complete run with a new annealing schedule which would need to be longer than the previous run. Therefore, SimWalk2 may need multiple runs with a large number of evaluations. However, in the EA, the convergence of the likelihood value can be checked at any time and any point during the run without any rescheduling or rerun. For convergence diagnosis shown in Figure 1, SimWalk2 needed to run more than 4 times because of rescheduling of the annealing process for each point (Figure 1). However, EA1 and EA2 needed only a single run for the same convergence assessment.

The EA population size was arbitrarily set at N = 10. The chance of operating EA-recombination or EA-mutation was randomly equal, i.e. the probability of EA-recombination (or EA-mutation) was ~0.5. Different EA-parameters did not dramatically affect the results unless the values were extreme, e.g. outside the range 4 – 30 for N or outside the range 0.25~0.75 for the probability of EA-recombination (or EA-mutation). If there are hundreds of CPUs available (possibly in the near future), then N should not be less than the number of CPUs used, to maximize the efficiency of parallel computing. Such large values for N for large scale parallel computing should be tested with regard to optimal performance. However, it is possible to set N considerably higher than optimal, and computational efficiency is still increased.

In the real data, we used 13 microsatellites on the same chromosome positioned at 12 cM intervals on average. However, a much larger number of markers is expected to be used soon, e.g. thousands of single nucleotide polymorphisms (SNP) at << ~1 cM intervals on a single chromosome. The EA with hundreds of CPUs may be able to parallelize and solve the increased size of the problem within a reasonable time. Haplotypes for dense markers might be resolved using population LD without pedigree information [22-24]. However, when population LD is not significant over the average marker distance, the methods may perform poorly [25]. Therefore, when LD is not sufficient or one may not be sure whether there is enough LD, our approach will be an efficient tool for haplotyping for most types of data.

When using multiple CPUs and multiple machines, data transfer between machines took a large part of the overall computing time. Therefore, the EA was slightly modified in order to save on transfer time. There were N nodes for EA-members parallelised between machines. We made each node have two EA-members (two sets of solutions). EA-mutation and EA-recombination were carried out in each node, and the solutions were evolved. One set of solutions in each node was moved to the next node (the order of the nodes was randomly determined) every *n *cycles, therefore a complete evolutionary mechanism was performed with periodic isolation of islands (nodes). The number for *n *was chosen as *n *= total number of iterations/*k*, which resulted in *k *transfers between nodes in an analysis. It is noted that *n *should be a sufficient number for EA-mutation and EA-recombination within each node, and *k *should be a sufficient number for communicating between the nodes. In this study, we chose a large number *k *= 800 which would be sufficient for *n *as well because we usually used the total number of iterations ≥ number of meiosis × number of markers × 800. Such a strategy becomes more important when using many different machines.

We used data sets having complex relationships with incomplete genotypes. Such data sets are quite common in natural and outbred populations [26]. Complex pedigrees with incomplete genotypes will generally generate a too large state space for haplotying, which cannot be handled by exact methods [8,9,27]. However, SimWalk2 has been successfully and widely used for such data sets although the size of data should be small or moderate. We showed that our approach could handle such data sets, and the computational efficiency for our approach was much higher than that for SimWalk2.

## Conclusion

The difference between EA and SA is mainly determined by the parameters used in their search mechanism, which affect the number of configurations considered in the cycles and updating strategies, and how information from different solutions is used to generate new candidate solutions. The EA algorithm has a substantial advantage in convergence assessment and parallel computing, which would much increase the efficiency of haplotype reconstruction (up to ~4 times with 4 CPU). Moreover, with the joint updating scheme, EA2 significantly outperformed SimWalk2 (up to ~8 times with 4 CPU). With more CPU, the computational efficiency of EA2 would be increased. In addition, our implementation of EA1 and EA2 for this application is likely to leave much room for increased performance, given the wide range of structural and parameterization strategies that could be invoked. Further study would be required to investigate such potential.

## Methods

### Distribution of segregation states given marker data and pedigree

A configuration of segregation states (*S*) given marker data in a pedigree can be expressed as an M × L matrix whose elements are 0 or 1, where M and L is the number of meiosis and marker loci, respectively. If the gene in the *m*^*th *^meiosis at the *l*^*th *^locus receives the paternal parental allele, the segregation indicator *S*_*ml *_= 0, and *S*_*ml *_= 1 for the maternal parental allele. The maximum number of possible configurations for *S *is 2^*M *× *L *^when none of pedigree members are genotyped. Probability of *S *given marker data in a pedigree can be written as,

(1)

where *G *represents the observed marker data, *pr(S) *is prior probability of the segregation indicators and haplotype configuration, *pr*(*G|S*) is the probability of the observed marker data given *S*, and the denominator is summed over the probabilities of all possible configurations of *S*.

### Haplotyping

There can be a large number of combinations for allele assignment to founder genes. A specific assignment of alleles to each founder gene combined with *S *defines the haplotype for each individual in the pedigree. The most likely haplotype configuration can be determined based on its likelihood [15,18].

### Likelihood estimation

The likelihood for observed marker data given a segregation state (*S*) and the most likely haplotype configuration given the segregation state (*H*_|*S*_) is a function of the probabilities of all recombinations in every meiosis and of the configuration of an ordered genotype assignment to the founders consistent with the segregation indicators.

(2)

where *S*_*ij *_is the segregation indicator for the *i*^*th *^meiosis at the *j*^*th *^locus, *θ*_*j *_is the recombination rate between marker *j *and *j+1 *and *g*_*k *_is the most probable founder genotype given the segregation state at the *k*^*th *^marker locus. Note that non-founder genotypes and haplotypes are totally dependent on the founder's genotypes and the segregation state at each marker. The computation of the first term in (2) is the function of all recombinations given *S *and is therefore relatively straightforward (|*S*_*ij*_*- S*_*ij*+1_| = 1 with recombination, and |*S*_*ij*_*- S*_*ij*+1_| = 0 with no recombination between *S*_*ij *_and *S*_*ij*+1_). For the second term, all possible genotype configurations for founders should be evaluated and the most likely configuration is chosen based on the likelihood given the segregation indicators (see [15,28]).

### SA for obtaining optimal haplotypes

Simulated annealing is a generic stochastic algorithm for optimization problems and it is analogous in operation to the annealing process in metallurgy [17]. There is one sequential haplotype configuration developed in consecutive iterations by temperature parameters with the Metropolis mechanism [29], applied to the random walk [15,19]. The probability of accepting a proposed value is given by

(3)

In the first stage of simulated annealing, the temperature is very high. Therefore any legal configuration can be broadly sampled. In the annealing process, the temperature gradually decreases and more likely configurations are more often taken than less likely configurations. The process reaches the lowest energy state that represents the optimal configuration [30,31].

### EA for obtaining optimal haplotypes

An evolutionary algorithm is a stochastic optimization algorithm based on the theory of evolution [20]. An EA-population consists of N individuals (or solutions), each representing a potential haplotype configuration, i.e. a possible *S *matrix. The parameters to be optimised by the EA are the individual segregation indicators. To generate a new solution in the next iteration, the parent is either mutated or recombined with another randomly chosen parent with 50% chance of each. EA-mutation follows 1/2^*t *^chance of *t *transitions of switching segregation indicators with the random walk [15]. EA-recombination follows EA-mutation, but a transition is executed by copying the *S *values from another randomly chosen solution, rather than switching states (0,1) under mutation, e.g. randomly chosen variables in one solution (e.g. i^th ^EA-individual) are substituted with those in another solution (e.g. j^th ^EA-individual). As an objective function, the likelihood of the haplotype set determined by the proposed variables from the EA-mutation or EA-recombination is evaluated (2). The EA-individual will acquire the updated variables if the new variables have a higher likelihood (EA-selection). With these processes in sequential steps, all EA-individuals are evolved toward the state of the global maximum.

### Joint updating schemes and the random walk for segregation indicators

In the process of updating segregation indicators, there are sets of variables that require simultaneous updating, i.e. the variables within the set are dependent on each other, therefore independent change of each variable is constrained by other variables in the set. However, it would be possible to find the set of dependent variables, tracing the change of allele assignments to founder gametes according to updated segregation indicators (Appendix). Then, joint updating for the set of dependent variables can be carried out, which will increase the acceptance rates for the proposal variables. Because of complex pedigree and genotype structure, not all sets of updated variables may be found. The random walk approach can be applied to those variables that have not been updated. This will make sure that the search covers all possible states, i.e. irreducibility.

### Initial legal configurations for N EA-members

Each EA-member requires a starting configuration, consistent with observed pedigree and marker data. The genotype elimination through inheritance constraint (GEIC) algorithm [32] is suitable for finding a legal configuration of segregation indicators at a single locus. This algorithm finds a legal configuration for each locus independently.

### Brief summary of the process

An evolutionary algorithm with the random walk approach alone (EA1), or an evolutionary algorithm with the joint updating method and the random walk approach (EA2) is briefly summarized below.

Obtaining starting configuration for each EA-individual

Do cycles

   *Do i = 1 ~ N EA-individuals*

      *Do j = 1 ~ no. meiosis × no. markers*

         *Joint updating (EA-mutation)*

         *Evaluate likelihood (EA-selection)*

      *End do ****(this loop is only for EA2)***

      *Do j = 1 ~ no. meiosis × no. markers × 10*

         *The random walk approach (EA-mutation or EA-recombination)*

         *Evaluate likelihood (EA-selection)*

      *End do ****(this loop is for both EA1 and EA2)***

   *End do*

   *Collect the updated solution from each EA-individual*

   *Evaluate the solution and save the best*

   *Convergence assessment*

End cycles

### Simulation study

An effective population size of 100 was simulated for 10 multiallelic marker loci for 100 generations to ensure that the population would have an equilibrium distribution of alleles at all loci after 100 generations. In each generation, the number of male and female parents was 50 and their alleles were transmitted to descendents on the basis of Mendelian segregation using the gene-dropping method [33].

The number of alleles assumed at each marker locus was 4 for multiallelic markers (e.g. microsatellites) and the starting allele frequencies were 0.25 in generation 0. The marker allele was mutated at a rate of 4 × 10^-4 ^per generation [34-36] where a new allele was added for mutated loci. Note that pedigree and genotype information was deemed not available for these 100 generations.

At generation 101, an effective population size of 20 was simulated for 5 generations with pedigree recording, and used for analysis. The total number of individuals used for analysis was 100. In each generation, the number of male and female candidate parents was 10 and they were randomly selected and mated such that the number of progeny for each parent was Poisson distributed with a mean of 2. Therefore, the recorded pedigree would have complex relationships between animals. Complete and incomplete genotypic data were simulated. In complete genotypic data, genotypes from all hundred pedigreed individuals were available for analysis. In incomplete genotypic data, genotypes were available for progeny in the last generation and ancestral and parental genotypes were all missing but their relationships were used.

### Real data

In addition to the simulation data, a real data set was also used. This consists of four half sib families with ancestral pedigree spanning approximately four to five generations. The four sires and some dams were related through the ancestral pedigree. Base animals were assumed unrelated. The number of individuals in this pedigree was 1010. The number in each half sib group varied from 50 to 200. The offspring were genotyped for 13 microsatellites on the same chromosome positioned at 12 cM intervals on average. However, ~40% of the genotypes were missing among the offspring. The ancestors and parents were not genotyped but their pedigree information was used. All individuals were related through complex relationships.

### Methods comparison

The performance of SimWalk2, EA1 and EA2 was compared. The number of evaluations of the likelihood function in each iteration round was one for SimWalk2, and N for EA1 and EA2. Therefore, the total number of likelihood evaluations is the same as the number of iteration in SimWalk2, and N times larger than the number of iterations in EA1 and EA2. N was set to 10. A parallel computing program, Parallel Virtual Machine (PVM) version 3 [37], was used for parallel computation of EA1 and EA2.

## Availability

A binary executable file for Linux operating system will be given on request.

## Appendix

### Methods for updating segregation indicators (SI)

**Figure 2:**
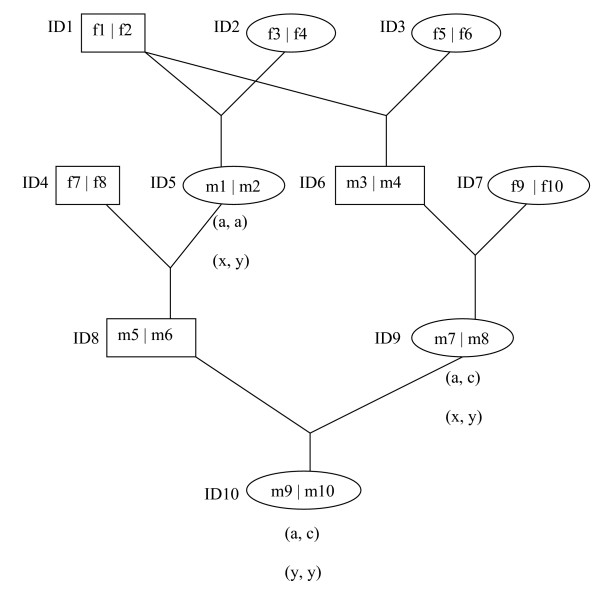
**Simple example pedigree**. Five founders with their numbered gametes (f1~ f10) and 5 descendents with their numbered meiosis (m1~m10). Unordered genotypes for the first and second marker are in the brackets underneath the animal ID5, ID9 and ID10 (other animals are not genotyped).

As a simple example, it is assumed that a pedigree has 5 founders and 5 descendents (Figure 2). There are 10 founder gametes (f1~f10) and 10 meioses (m1~m10). The animal ID5, ID9 and ID10 are genotyped for two markers, e.g. unordered genotypes for ID5, ID9 and ID10 are (a, a), (a, c) and (a, c) for the first marker and (x, y), (x, y) and (y, y) for the second marker (Figure 2). Each marker has 3 alleles coded as a, b and c for the first marker and x, y and z for the second marker. The population frequency for a, b and c is 0.3, 0.2 and 0.5 in the first marker, and that for x, y and z is 0.1, 0.2 and 0.7 in the second marker. The distance between two markers is 10 cM (recombination rate = ~0.09).

A legal configuration of SI for the meioses (from m1 to m10) and allele assignment to the founder gametes (from f1 to f10) are shown for each marker in Table 3. The allele assignments for the founder gametes are derived from the SI and genotypic data (see [15,28,38]). In equation (2), the first term in the right hand side can be calculated from the SI, and the second term can be calculated from the allele assignments to the founder gametes (for non-informative founder gametes (-), the most frequent allele can be assigned for maximizing the likelihood). The log likelihood is -46.143.

**Table 3:** A legal configuration of segregation indicators for the meiosis, and allele assignment to the founder gametes given the segregation indicators at the first and second marker

	m1	m2	m3	m4	m5	m6	m7	m8	m9	m10
marker 1	0	1	0	1	0	1	1	0	1	0
marker 2	1	0	1	0	1	0	0	0	0	0

	f1	f2	f3	f4	f5	f6	f7	f8	f9	f10

marker 1	a	-	-	a	-	c	-	-	a	-
marker 2	-	y	x	-	-	-	-	y	x	-

Log likelihood = -46.143

### Single site updating

This strategy switches segregation indicator for a meiosis at a marker locus (single site) subsequently, with the likelihood evaluation to lead to a state with higher likelihood. Table 4 shows the SI updated site by site with the likelihood evaluation. Switching the first meiosis (m1) at the first marker results to a different set of allele assignments for the founder gametes, and the number of recombination events decreases (Table 4I). This gives an improved likelihood (-43.837) and the first meiosis at the first marker is updated. Subsequently, the second meiosis is switched and the calculated likelihood is -41.531 (Table 4II). It is noted that the switched site is updated only when the likelihood improves. The following meiosis is subsequently switched and evaluated in the same manner. The SI, the allele assignments to the founder gametes given the SI, and the calculated likelihood is shown in Table 4 (III, IV, V and VI for m3, m4, m5 and m6 at the first marker). However, when m7 at the first marker is switched from 1 to 0, two different alleles are simultaneously assigned to the same founder gamete (f2), which is an illegal state (Table 4VII).

**Table 4:** Updating segregation indicators for a meiosis at a marker subsequently, and the change of allele assignment to the founder gamete given the updated segregation indicators

I. Log likelihood = -43.837										
	m1	m2	m3	m4	m5	m6	m7	m8	m9	m10

Marker 1	**1**	1	0	1	0	1	1	0	1	0
Marker 2	1	0	1	0	1	0	0	0	0	0

	f1	f2	f3	f4	f5	f6	f7	f8	f9	f10

Marker 1	-	**a**	-	a	-	c	-	-	a	-
Marker 2	-	y	x	-	-	-	-	y	x	-

II. Log likelihood = -41.531										

	m1	m2	m3	m4	m5	m6	m7	m8	m9	m10

marker 1	1	**0**	0	1	0	1	1	0	1	0
marker 2	1	0	1	0	1	0	0	0	0	0

	f1	f2	f3	f4	f5	f6	f7	f8	f9	f10

marker 1	-	a	**a**	-	-	c	-	-	a	-
marker 2	-	y	x	-	-	-	-	y	x	-

III. Log likelihood = -39.225										

	m1	m2	m3	m4	m5	m6	m7	m8	m9	m10

marker 1	1	0	**1**	1	0	1	1	0	1	0
marker 2	1	0	1	0	1	0	0	0	0	0

	f1	f2	f3	f4	f5	f6	f7	f8	f9	f10

marker 1	-	a	a	-	-	c	-	-	a	-
marker 2	-	y	x	-	-	-	-	y	x	-

IV. Log likelihood = -36.919										

	m1	m2	m3	m4	m5	m6	m7	m8	m9	m10

marker 1	1	0	1	**0**	0	1	1	0	1	0
marker 2	1	0	1	0	1	0	0	0	0	0

	f1	f2	f3	f4	f5	f6	f7	f8	f9	f10

marker 1	-	a	a	-	**c**	-	-	-	a	-
marker 2	-	y	x	-	-	-	-	y	x	-

V. Log likelihood = -34.614										

	m1	m2	m3	m4	m5	m6	m7	m8	m9	m10
marker 1	1	0	1	0	**1**	1	1	0	1	0
marker 2	1	0	1	0	1	0	0	0	0	0

	f1	f2	f3	f4	f5	f6	f7	f8	f9	f10

marker 1	-	a	a	-	c	-	-	-	a	-
marker 2	-	y	x	-	-	-	-	y	x	-

VI. Log likelihood = -32.308										

	m1	m2	m3	m4	m5	m6	m7	m8	m9	m10

marker 1	1	0	1	0	1	**0**	1	0	1	0
marker 2	1	0	1	0	1	0	0	0	0	0

	f1	f2	f3	f4	f5	f6	f7	f8	f9	f10

marker 1	-	a	a	-	c	-	-	-	a	-
marker 2	-	y	x	-	-	-	-	y	x	-

VII. State is illegal										

	m1	m2	m3	m4	m5	m6	m7	m8	m9	m10

marker 1	1	0	1	0	1	0	**0**	0	1	0
marker 2	1	0	1	0	1	0	0	0	0	0

	f1	f2	f3	f4	f5	f6	f7	f8	f9	f10

marker 1	-	**a and c**	a	-	-	-	-	-	**a or c**	-
marker 2	-	y	x	-	-	-	-	y	x	-

### Joint update with dependent variables

Table 5 shows the segregation state of founder gametes for genotyped animals and the changes due to switching m7 at the first marker. After switching the m7 (Table 4VII), the founder gamete 5 is changed to the founder gamete 2 for the paternal allele of the animal 9 and the maternal allele of the animal 10. However, the founder gamete 2 is already assigned to the paternal allele of the ID10, therefore, two different alleles (a and c) have to be simultaneously assigned to the founder gamete 2 (illegal state). In order to avoid an illegal state, the paternal gamete for ID10 should be simultaneously changed to other legal founder gametes. For this, three sets of joint updating can be found, i.e. joint updating of m1 and m7, m7 and m9, and m7 and m10 (Table 6).

**Table 5:** Segregation state of founder gametes for genotyped animals and the changes due to switching m7 at the first marker

Genotyped animal	paternal	maternal
ID5	**f2**	f3
ID9	F5 -> f2	f9
ID10	**f2**	F5 -> f2

**Table 6:** Joint updates

With joint update for m1 and m7 (log likelihood = -32.308)										
	m1	m2	m3	m4	m5	m6	m7	m8	m9	m10

Marker 1	**0**	0	1	0	1	0	**0**	0	1	0
Marker 2	1	0	1	0	1	0	0	0	0	0

	f1	f2	f3	f4	f5	f6	f7	f8	f9	f10

marker 1	**a**	**c**	a	-	-	-	-	-	a	-
marker 2	-	y	x	-	-	-	-	y	x	-

With joint update for m7 and m9 (log likelihood = -27.185)										

	m1	m2	m3	m4	m5	m6	m7	m8	m9	m10

marker 1	1	0	1	0	1	0	**0**	0	**0**	0
marker 2	1	0	1	0	1	0	0	0	0	0

	f1	f2	f3	f4	f5	f6	f7	f8	f9	f10

marker 1	-	a	a	-	-	-	-	**c**	**c**	-
marker 2	-	y	x	-	-	-	-	y	x	-

With joint update for m7 and m10 (log likelihood = -31.797)										

	m1	m2	m3	m4	m5	m6	m7	m8	m9	m10

marker 1	1	0	1	0	1	0	**0**	0	1	**1**
marker 2	1	0	1	0	1	0	0	0	0	0

	f1	f2	f3	f4	f5	f6	f7	f8	f9	f10

marker 1	-	a	a	-	-	-	-	-	**c**	-
marker 2	-	y	x	-	-	-	-	y	x	-

## Authors' contributions

SHL conceived the idea, developed the EA algorithm with a computer program and carried out the analyses with simulated and real data, and drafted the manuscript. JVDW contributed to study design and manuscript preparation, and supervised all experiments. BK contributed to coordination of the EA part of the work, and manuscript preparation. All authors read and approved the final manuscript.

## Supplementary Material

Additional file 1 Results for 10 replicates for simulated data Click here for file
